# Supplementary material for: Species phylogenetics using intraprotein residue dependence inferred with protein foundation models
Source: bioRxiv. 2026 Oct 27:2026.05.26.727927. Preprint. [Version 2] doi: 10.64898/2026.05.26.727927 (PMC13733735; doi:10.64898/2026.05.26.727927)

bioRxiv preprint doi: https://doi.org/10.64898/2026.05.26.727927; this version posted October 27, 2026. The copyright holder for this preprint (which was not certified by peer review) is the author/funder, who has granted bioRxiv a license to display the preprint in perpetuity. It is made available under a CC-BY 4.0 International license.

**Supplementary Figure S1: Computational runtime for pFM tree-building.** Computational time required for single-threaded *RAM*, *SAM*, and ML analysis on a personal computer with 64 GB of RAM and 32 processors.

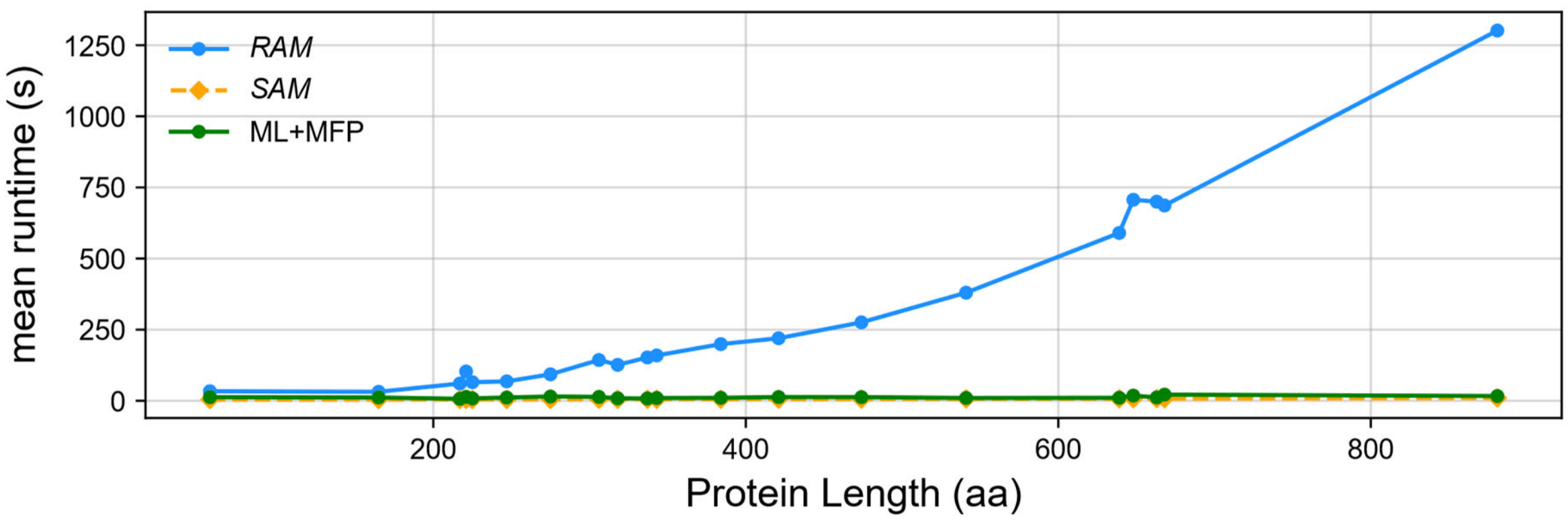

Supplement: Supplementary file 1 [file NIHPP2026.05.26.727927v2-supplement-2.pdf]
